# POSITIVE EFFECTS OF LOWER EXTREMITY CONSTRAINT-INDUCED MOVEMENT THERAPY ON BALANCE, LEG STRENGTH AND DUAL-TASK ABILITY IN STROKE PATIENTS: A LONGITUDINAL COHORT STUDY

**DOI:** 10.2340/jrm.v56.24168

**Published:** 2024-10-03

**Authors:** Annika SEFASTSSON, Ingela MARKLUND, Håkan LITTBRAND, Per WESTER, Britt-Marie STÅLNACKE, Ann SÖRLIN, Birgitta LANGHAMMER, Per LIV, Xiaolei HU

**Affiliations:** 1 Department of Community Medicine and Rehabilitation, Rehabilitation medicine, Umeå University, Umeå; 2 Liljeholmskliniken, Stockholm; 3 Centre for Clinical Research and Education, Region Värmland, Karlstad; 4 Department of Community and Rehabilitation Medicine, Geriatric Medicine, Umeå University, Umeå; 5 Department of Public Health and Clinical Medicine, Umeå University, Umeå; 6 Department of Clinical Science, Karolinska Institute Danderyds Hospital, Stockholm, Sweden; 7 Faculty of Health Sciences, Oslo Metropolitan University, Oslo, Norway

**Keywords:** balance, constraint-induced movement therapy, dual-task, high-intensity training, lower extremity, physical therapy, strength training, stroke rehabilitation

## Abstract

**Objective:**

To investigate whether high-intensity lower extremity constraint-induced movement therapy can improve balance, leg strength, and dual-task ability.

**Design:**

A longitudinal cohort study in a real-world outpatient clinic.

**Patients:**

147 community-dwelling participants in the subacute and chronic poststroke phases.

**Methods:**

Participants received lower extremity constraint-induced movement therapy for 6 hours/day during 2 consecutive weeks, including balance, strength, and functional training. The Berg Balance Scale (BBS), Single-Leg-Stance (SLS) bilaterally, one Repetition Maximum (1RM) in a leg press, symmetry of leg strength (Diff-1RM), Timed Up and Go (TUG), and the TUG Manual test were assessed before, after, and 3 months after lower extremity constraint-induced movement therapy.

**Results:**

Compared with preintervention data, statistically significant improvements after lower extremity constraint-induced movement therapy (*p* < 0.001) were demonstrated for balance with an absolute value in BBS at 1.9 points (effect size 0.38) and SLS at 2.4 s (effect size 0.24), and for leg strength at 10.2 kg (effect size 0.54) for the affect-ed leg. Diff 1RM decreased significantly at 5.8 kg (effect size 0.39) and improvements on dual-task ability at 2.7 s were significant (effect size 0.14). The effects persisted at the 3-month follow-up.

**Conclusions:**

High-intensity lower extremity constraint-induced movement therapy may be a feasible treatment option for middle-aged stroke patients to affect balance, leg strength, and dual-task ability positively in an out-patient clinical setting.

## LAY ABSTRACT

Despite convincing evidence of the effectiveness of high-intensity upper extremity constraint-induced movement therapy in research settings and recommendations in Swedish national stroke guidelines, clinical implementation of the therapy has so far been limited. Similar issues have been addressed regarding lower extremity constraint-induced movement therapy. It is thus interesting to evaluate whether lower extremity constraint-induced movement therapy may be adopted in a real-world setting, as randomized controlled trials have demonstrated positive effects of lower extremity constraint-induced movement therapy. Compared with preintervention data, balance, leg strength ,and dual-task ability improved significantly, with low and moderate effect sizes, after lower extremity constraint-induced movement therapy among 147 community-dwelling participants aged around 50 in the subacute and chronic phases after stroke. Improvements persisted at the 3-month follow-up after lower extremity constraint-induced movement therapy despite diverse backgrounds such as sex, type of stroke, affected side, and time since stroke onset. High-intensity lower extremity constraint-induced movement therapy may thus be a feasible and useful treatment option to affect balance, leg strength, and dual-task ability positively post stroke.

Balance deficits and muscle weakness are common physical impairments, with a negative impact on activity and participation levels following a stroke (1, 2). Impaired balance and muscle weakness, as well as asymmetric strength of lower extremities (2, 3) and reduced dual-task capacity (4), have all been identified as important risk factors for falls (5). Balance and strength impairments, in addition to an expected continuous decrease of muscle strength in chronic stroke survivors, is likely to accelerate the progression of disability (4). Hence, an important focus and immense challenge in stroke rehabilitation is to improve balance, strength, and functional mobility in early and chronic stages post-stroke.

Various physical therapy strategies are being used to enhance balance and strength in stroke survivors (6, 7). Constraint-induced movement therapy (CIMT) is a treatment modality initially developed to enhance motor function of impaired upper extremities (UE) after central nervous system (CNS) damage (8, 9). Based on positive results from UE-CIMT (8, 10), the UE protocol was eventually modified into a LE-CIMT protocol including the cornerstones: (*i*) intensive supervised training, (*ii*) motor training based on shaping strategy, (*iii*) application of a transfer package, (*iv*) strongly encouraging use of the more-affected lower extremity (11). These cornerstones have partially been adopted in various other physical therapy approaches, such as high-intensity training, task-specific-training, and musculoskeletal intervention, with mostly positive effects on gait, balance, and strength among persons with stroke (7, 12).

Despite convincing evidence of the effectiveness of UE-CIMT in research settings (8, 10) and recommendations in some national clinical guidelines, such as in Sweden (13), clinical implementation has thus far been limited (14). This may mainly be due to perceived problems with generalizability (limited target group), high resource demands, and concerns about patient tolerance as well as insufficient knowledge and confidence in CIMT’s effectiveness (14). Similar issues have been addressed regarding LE-CIMT (12, 15, 16). Studies have been carried out with a wide variety of designs and levels of intensity. Different approximate definitions are being used such as high-intensity modified CIMT (3–6 h/day) and low-intensity modified CIMT (<3 h training/day) (17). In the present study, we used the original high-intensity CIMT with 6 h training/day. It is thus interesting to evaluate whether LE-CIMT may be adopted in a real-world setting with equally positive results, because randomized controlled trials (RCTs) have demonstrated improved balance and ambulation (15, 18–20).

We have previously shown that LE-CIMT in a real-world setting may improve motor function, mobility, and walking after stroke (21). However, less is known about how LE-CIMT affects balance, leg strength and the ability to perform dual tasks. The primary objective of the current study was to investigate whether LE-CIMT can improve balance, lower-limb strength, and dual-task ability after stroke. Second, we aimed to evaluate whether the effect of the therapy on balance was affected by various demographic and clinical characteristics, such as age, sex, type of stroke, affected side, or time since stroke onset.

## MATERIALS AND METHODS

### Study design

This longitudinal cohort study was carried out in an outpatient clinic in Stockholm, Sweden, between 2003 and 2018. The clinic offered upper and lower extremity CIMT for patients with hemiplegia after CNS damage. Prior to entering the study, patients had undergone rehabilitation of differing scope according to current clinical routines and were mainly referred to the outpatient clinic from different hospital clinics in the Stockholm area.

Ethical approval was obtained from the regional Ethical Review Board in Umeå and, as of 2019, the Swedish Ethical Review Authority, Sweden (Dnr. 2013-327-31 M).

Data from patients who had received LE-CIMT at the clinic between 2003 and 2014 were collected retrospectively from medical records and thereafter prospectively until 2018 with the same study protocol. Participants were followed prospectively and provided written informed consent prior to inclusion. This study conformed to the Helsinki declaration.

### Inclusion criteria

The inclusion criteria were: (*i*) adults with a first-ever stroke, (*ii*) ability to walk indoors without a walking aid, (*iii*) ability to understand verbal and/or written instructions, (*iv*) had not previously received LE-CIMT (i.e. 6 h/day for 2 weeks). Patients with an unstable cardiovascular condition were excluded at referral.

### Intervention

Participants received individually tailored LE-CIMT in groups of 4 participants. Treatment followed the main principles of the LE-CIMT protocol (11):

*(i) Intensive supervised training.* The intervention was intensive massed practice (6 h/day for 10 consecutive weekdays) supervised by a physiotherapist (PT) (the first author) who had extensive clinical experience with CIMT.

The LE-CIMT included various training sessions focusing on balance, strength, locomotor training indoors and outdoors, functional training, and stretching of short muscles. Training programmes were based on pre-test results and individual goals and were revised on a daily basis by the PT. The proportion of task-specific exercises and impairment-focused training varied depending on the individual’s needs. Whenever appropriate, the emphasis was on task-specific exercises. Balance was practised with a variety of support surfaces, such as the floor and different foam balance cushions and positions of feet, combined with diminishing available hand support and changing performance speed. The Wii balance board was used. In addition, balance- and strength-enhancing functional exercises were performed, such as standing up/sitting down and picking up objects from low stools or floor. Strength exercises addressing both maximal (80% of 1RM 6 x 6 repetitions) and enduring strength (60% of 1RM 5 x 15–20 repetitions) were performed in a leg press with a daily increase of resistance of approximately 5%. Emphasis was also put on strength and coordination of plantar- and dorsiflexion of the ankle and on mobility and strength of feet including toes. Walking with a constraining splint on the stronger leg to hinder knee flexion and thereby increasing weight-bearing load on the weaker leg during the swing phase of the stronger leg was used for periods of time during the day. Frequent feedback was given by the PT throughout sessions to ensure intervention fidelity with good quality regarding performance. Exercises were chosen to maximize use of the weaker leg, such as doing strength exercises in the leg press with merely the weaker leg or unilateral pedalling on a bicycle. Patients were given an individual home exercise programme of approximately 1 h during the 2 weekends included in the treatment period.

*(II) Motor training based on shaping strategy.* According to the shaping strategy, more complex activities were practised in small steps at a time, with a gradually increasing level of difficulty to ensure that goals seemed achievable to the individual. The achieved skills were eventually assembled into a more complete activity, which could be expressed as “practising parts” before “practising tasks” (22). An example is walking up and down stairs, which could typically involve strength training of leg muscles, including hip and foot, stretching of short calf muscles, and step-up exercises with increased load, speed, and height to enhance the ability to practise the activity itself in a normalized manner. The shaping strategy demands a thorough analysis of patient deficits.

*(III) Application of a transfer package.* After the period of LE-CIMT, the participants received an individual programme that they were recommended to do at home until the 3-month follow-up. The content and the frequency with which to perform the programme was decided in collaboration with the individual. Participants were encouraged to keep a simple diary of the extent of training performed. There was merely an oral follow-up on compliance with the home exercise programme. Patients could contact the PT for consultation if needed from the end of treatment until follow-up.

*(IV) Strongly encouraging use of the affected lower extremity*. General instructions for the treatment period were given orally and in writing as follows: (*i*) to put more weight on weak leg when standing; (*ii*) to place the weaker foot closer to the chair than the stronger foot when getting up and sitting down to enable more weightbearing on the weaker leg; (*iii*) to walk up flights of stairs with the weaker foot first and down stairs with the stronger foot first to put more strain on the weaker leg; (*iv*) to avoid helping out with the stronger leg or hand when flexing the weaker knee in sitting position or when lifting the leg into bed or crossing the legs. Instructions were to try 3 times before helping out with the stronger side.

### Outcome measures

Participants were assessed before and after treatment and at the 3-month follow-up. Outcome measures were the Berg Balance Scale (BBS), single-leg-stance (SLS) bilaterally, 1 repetition maximum (1RM) of leg extension in a leg press, timed up and go (TUG), and TUG Manual (carrying a glass of water while performing the TUG).

The assessments were performed by a PT (the first author), which is in accordance with normal clinical practice. The charts from previous assessments were not available to the PT at retest. Tests were performed according to a written protocol to ensure that patient instructions were given in a standardized manner and tests were administered in the same order. No verbal encouragement was given during testing. Patients could wear their ankle/foot orthosis but not employ their normally used walking aid.

*Berg Balance Scale*. The BBS was used to assess functional balance capacity. The scale consists of 14 items rating the participant’s ability to maintain stability while performing a functional task on a 5-point (0 to 4) scale with a maximum score of 56 points (23). The BBS has been proven to be highly valid and reliable in the stroke population. The minimal detectable change (MDC) for BBS has been calculated at 2.7 points for chronic stroke (24) and 4 points in the early subacute stage for patients scoring 45–56 points and walking without assistance at study admission (31), while the minimal clinically important change (MCID) is estimated to be 4–5 points in the early subacute phase (25).

*Single-leg-stance.* SLS (26) was tested bilaterally, twice for each leg for a maximum of 30 s, and the best result for each leg was included in the analyses. The test assesses static postural control. MDC for SLS is 24.1 s in community-dwelling individuals aged 60 years or older (27).

*Leg strength as 1 repetition maximum (1RM) and leg strength symmetry (Diff-1RM)*. Lower-limb strength was measured bilaterally as 1 repetition maximum (1RM) for leg extension in a leg press machine (28). 1RM is defined as the maximum weight a person can lift once before becoming fatigued. The nonaffected leg was tested first. The starting load for the affected leg was set at approximately 50% of the 1RM of the nonaffected leg the first time. The ambition was to perform as few trials as possible to establish 1RM. Weight intervals of 1 kg were used. The rest between each attempt was 30 s. The difference in leg strength between the affected and nonaffected leg (Diff-1RM) illustrates the degree of leg strength symmetry. The difference in leg strength between legs was calculated as the ratio between affected limb/nonaffected limb.

*Timed Up and Go, Timed Up and Go Manual and DiffTUG*. The TUG assesses functional mobility and balance. It is a commonly used test in the stroke population and has shown good reliability (4). To perform the TUG, an individual is timed while rising from an armrest chair, walking 3 m, turning around, returning to the chair, and sitting down.

The TUG Manual is a development of the TUG. A manual task, carrying a glass of water, is added to the TUG procedure (29). An inability to perform dual tasks may be an indicator of an increased risk of falls (30). A timed difference of >4.5 s between performing the TUG and the TUG Manual predicts a higher risk of falling with community-dwelling older people aged around 80 (29). MDC for TUG is calculated to be 3.2 s in the chronic stage post-stroke (24)

### Data presentations and statistical analyses

Demographic characteristics are presented as the mean (SD), number with/without number of cases (%) or the difference between 2 means with 95% confidence interval (CI) when appropriate. The time span from stroke onset to LE-CIMT intervention varied from 1 month to more than 10 years. Participants were categorized into 3 subgroups depending on the time between stroke onset and treatment intervention: 1–6 months (subacute phase), 7–12 months (chronic phase), and ≥ 13 months (late chronic phase) post-stroke.

The effect sizes (EF) were calculated as = (mean of postintervention – mean of pre-intervention)/SD (pre-intervention). According to Cohen’s d, 0.2 is considered a small effect size, 0.5 is a moderate effect size, and 0.8 is considered a large effect size (31).

A linear mixed model (LMM) was used to investigate mean differences between measurements pre- and post-treatment and at follow-up. An unstructured covariance matrix was used to model dependence between residuals, and time was considered a fixed categorical effect. Post hoc tests were performed on marginal means using Bonferroni correction of p-values to account for multiple comparisons between the time points. Q‒Q plots were used to verify the normal distribution assumption for model residuals.

Furthermore, to examine heterogeneity regarding the effect on BBS, we fitted additional corresponding LMMs by including age, gender, type of stroke, affected side, or time to intervention as main fixed effects as well as their interactions with time.

All data were analysed using the Statistical Package for the Social Sciences (SPSS) version 26.0 Software for Windows (IBM Corp, Armonk, NY, USA). The figures were generated by GraphPad Prism 9 (San Diego, CA, USA). A two-tailed *p*-value < 0.05 was considered significant.

### Data availability

The data associated with the paper are not publicly available but are available from the corresponding author on reasonable request.

## RESULTS

### Participants

**Fig. 1:**
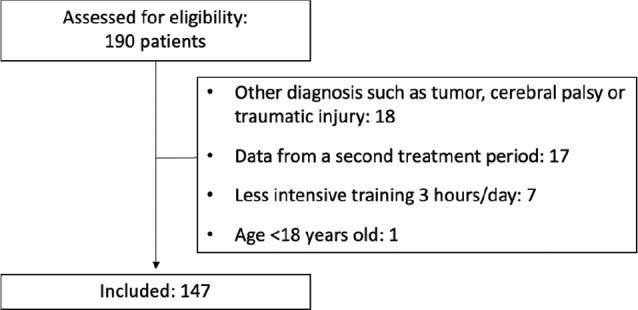
Flowchart of participant recruitment.

A total of 190 patients underwent LE-CIMT at the clinic and were assessed for study eligibility. A total of 147 subjects fulfilled the inclusion criteria and were included in the study (Fig. 1).

### Basic demographic and clinical characteristics

The baseline characteristics of the study participants in total and divided into subgroups depending on the time between stroke onset and LE-CIMT intervention are presented in Table I. The mean age of the participants was 50.7 (SD 10.6) years at stroke onset. One hundred participants (68%) were male. Half of the participants suffered an ischaemic stroke (51%), while one-third had a haemorrhagic stroke. More than half of the participants (57%) had right-sided hemiplegia. The basic demographic characteristics showed similar patterns in the 3 subgroups at different phases after stroke onset. The time span from stroke onset until receiving LE-CIMT varied, with a mean of 18 (SD 25) months.

**Table I:** Demographic and clinical characteristics

Patient characteristics	Total # (*n* = 147)	1–6 months after stroke (*n* = 29)	7–12 months after stroke (*n* = 59)	>13 months after stroke (*n* = 58)
Age, mean (SD)	50.7 (10.6)	49.5 (11.9)	52.6 (10.0)	49.5 (10.3)
Gender, male/female, *n*	100/47	20/9	42/17	37/21
Type of stroke, infarction/haemorrhage/not specified, *n*	75/50/22	14/10/5	32/19/8	29/21/8
Affected lower extremity, right/left, *n*	84/62	18/11	34/25	32/26
Time to LE-CIMT after stroke, months, mean (SD)	18 (25)	4.2 (1.5)	9.4 (1.8)	32.5 (33.7)

A total of 147 participants categorized in subgroups based on time after stroke onset, i.e., 1–6 months (subacute phase), 7–12 months (chronic phase) and ≥ 13 months (late chronic phase) after stroke onset.

# missing value on the time span between LE-CIMT and stroke onset for 1 participant.

The participants’ compliance with the intervention was almost 100%, as participants were informed beforehand that full participation was demanded.

However, 40 participants did not turn up for the 3-month follow-up. A dropout analysis in our previous study (21) showed no statistically significant differences in descriptive characteristics between these 40 patients and the patients who completed all 3 measurement occasions.

### Improvements in balance

**Fig. 2:**
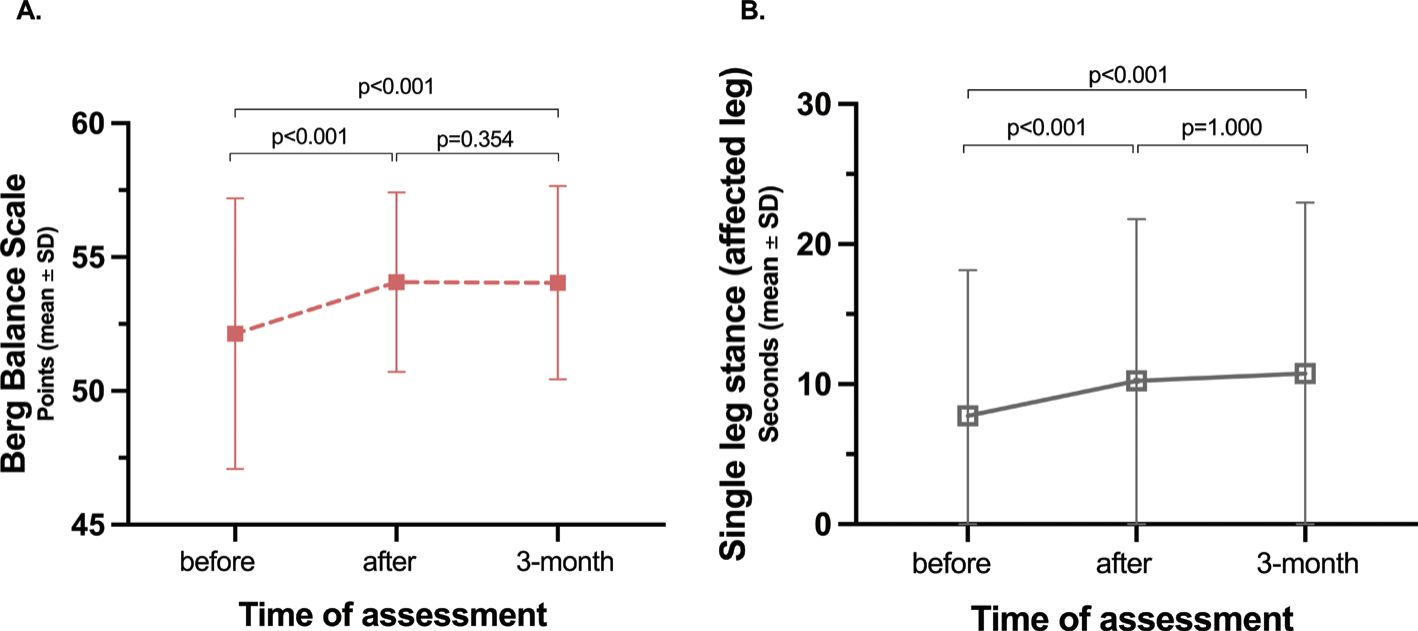
Improvements in balance assessed by the Berg Balance Scale (A) and single leg stance (B).

In this cohort, 40% of the participants reached the maximum score of 56, and a total of 50% of the participants tested at 55–56 points on the BBS at the preintervention assessment. Compared with the assessment prior to treatment, the BBS score demonstrated statistically significant improvements after LE-CIMT, which persisted at the 3-month follow-up. The mean difference in preintervention BBS scores compared with postintervention scores was 1.9 points (CI 1.3–2.4, *p* < 0.001, EF 0.38) and 1.7 points (CI 1.1–2.3, *p* < 0.001, EF 0.38) at follow-up (Fig. 2A).

Timed SLS for the affected leg improved significantly, by 2.4 s after LE-CIMT compared with baseline (CI 0.9–3.8, p < 0.001, EF 0.24), with enhancement remaining at follow-up (CI 0.8–3.8, p < 0.001, EF 0.29) (Fig. 2B). No significant improvements were seen for the nonaffected leg post-treatment with mean difference being 1.2 s (CI at 0.3–2.7, p = 0.19, EF 0.12) or at follow-up with mean difference being 1.0 s (CI at 0.7–2.7, p = 0.51, EF 0.11).

### Improvements in leg strength and leg strength symmetry

**Fig. 3:**
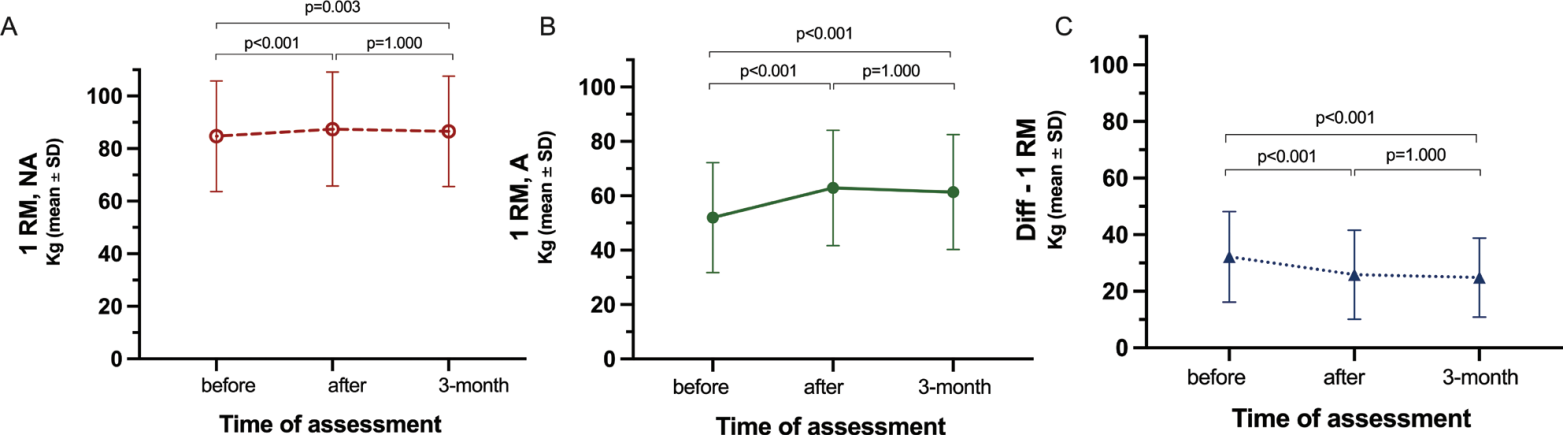
Improvements in leg strength for the (A) nonaffected and (B) affected leg and (C) the difference in leg strength between the nonaffected and affected legs.

Leg strength (1RM) demonstrated statistically significant enhancement in the nonaffected and affected leg after and at follow-up compared with before the intervention (Fig. 3). For the nonaffected leg, the mean difference between preintervention and postintervention was 3.5 kg (CI 1.5–5.6, *p* < 0.001, EF 0.13) and 3.9 kg (CI 1.1–6.7, *p* =0.003, EF 0.09) for the follow-up scores (Fig. 3A). This corresponded to 4% and 5% increase in leg strength, respectively. For the affected leg the mean difference for preintervention 1RM compared with postintervention was 10.2 kg (CI 8.6 to 11.8, *p* < 0.001, EF 0.54) and 10.1 kg (CI 7.7 to 12.6, *p* < 0.001, EF 0.47) at follow-up (Fig. 3B). This corresponded to a 19% strength increase in the affected leg.

There was no significant difference between postintervention and the 3-month follow-up for either nonaffected or affected leg (Fig. 3A and 3B).

There was a statistically significant decrease in the difference in leg strength between legs after LE-CIMT remaining at follow-up (Fig. 3C). The mean difference for preintervention Diff-1RM compared with postintervention was –5.8 kg (CI –8.1 to –3.5, *p* < 0.001, EF 0.39) and – 6.0 kg (CI –9.0 to –3.0, *p* < 0.001, EF 0.46) for postintervention compared with follow-up (Fig. 3C). The results demonstrate a decrease in leg strength asymmetry of approximately 20% after LE-CIMT. The ratio between affected/nonaffected limb of 0.61 at pre-intervention increased to 0.72 at post-intervention and 0.71 at follow-up.

### Improvements in dual-task ability

**Fig. 4:**
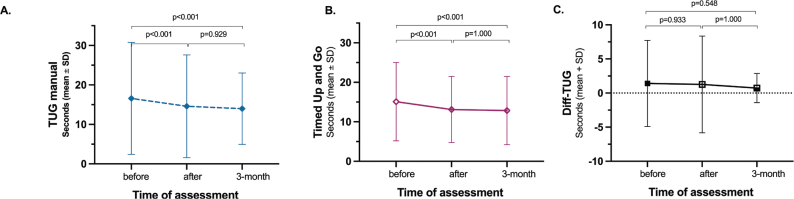
Results for dual task assessed by the (A) TUG Manual test compared with (B) TUG test.

The time to perform the TUG manual showed statistically significant reductions after and at the 3-month follow-up compared with before the intervention (Fig. 4A). The TUG manual scores improved by 2.7 s (CI 1.6 to 3.7, *p* < 0.001, EF 0.14) from pre- to postintervention and by 2.9 s (CI 1.7 to 4.1, *p* < 0.001, EF 0.18) at the 3-month follow-up compared with preintervention.

Compared with the pre-test, as demonstrated previously (21), the TUG score improved by 2.5 s (CI 1.3–3.7, *p* < 0.001, EF 0.20) at post-test and 2.8 s (CI 1.3–4.3, *p* < 0.001, EF 0.22) at follow-up (Fig. 4B). No significant change was seen on the Diff-TUG (Fig. 4C).

### Heterogeneity analysis for the BBS over time

Most of the demographic and clinical characteristics, including sex (*p* = 0.75), type of stroke (*p* = 0.31), affected side (*p* = 0.33), and time span between stroke onset and receiving LE-CIMT (*p* = 0.97), did not demonstrate any significant interactions with BBS over time. However, the younger participants showed significantly higher BBS scores (*p* = 0.02) than the older participants in this middle-aged cohort.

### Similar improvements in the 3 subgroups based on time after stroke onset

Similar to the cohort as a whole, most subgroups showed statistically significant improvements post-treatment and at follow-up for dual-task ability (TUG manual), symmetric leg strength (Diff 1RM), and mostly for balance (BBS) (Table II). No statistically significant difference was observed between different subgroups for either outcome.

**Table II:** Subgroup analyses on balance, leg strength, and dual-task ability

Outcomes (mean (SD))	Total (*n* = 147) #	1-6 months after stroke (*n* = 29)	7-12 months after stroke (*n* = 59)	> 13 months After stroke (*n* = 58)
**BBS (points)**				
before	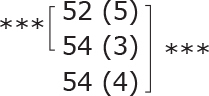	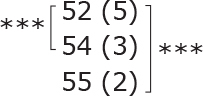	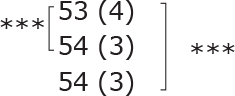	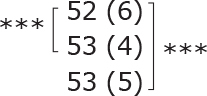
after				
3-month after				
**Diff-1 RM (kg)**				
before	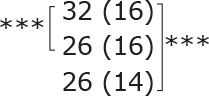	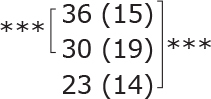	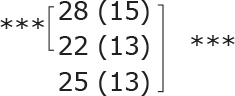	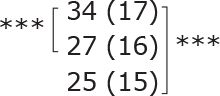
after				
3-month after				
**TUG manual (seconds)**				
before	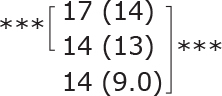	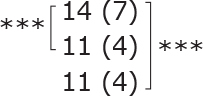	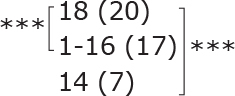	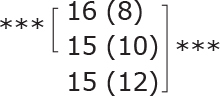
after				
3-month after				

There were no significant differences in outcomes between the different subgroups when LE-CIMT was performed at 1–6 months (subacute phase), 7–12 months (chronic phase) and ≥ 13 months (late chronic phase) after stroke onset. The marginal mean estimates from the model.

# missing value on the time between LE-CIMT and stroke onset for 1 participant.

***p < 0.001,

** *p* < 0.01,

*p < 0.05.

## DISCUSSION

In the current study, we investigated the impact of high-intensity LE-CIMT on balance, lower-limb strength, and dual-task ability among 147 persons after stroke in a real-world outpatient clinical setting. At postintervention, statistically significant improvements with small to moderate EF were seen regarding balance, lower-limb strength, leg strength symmetry and dual-task ability. The positive effects persisted at the 3-month follow-up. Participant characteristics, such as sex, type of stroke, affected side, and time span between stroke onset and treatment, did not influence the effects on balance assessed by BBS. However, younger participants showed better balance than older participants in this middle-aged cohort.

The significant statistical improvements with an absolute value of 1.9 points on the BBS in this study indicated that LE-CIMT affected balance positively among persons with stroke in the chronic stage. Our data are in line with previous randomized controlled trials (RCTs) (19, 20) and systematic reviews (6) where improvements on the BBS ranging between 1.26 and 3.17 points were demonstrated in the subacute and chronic phases post-stroke. Furthermore, the EF on the BBS (0.38) in our study is similar to the results in a recent well-defined RCT where balance was assessed by the mini-Balance Evaluation System Test (mini-BESTest) with less of a ceiling effect (20). In the future, the mini-BESTest could be a preferable alternative to minimize the ceiling effect of BBS in a research setting as well as in daily clinical practice. To counteract the ceiling effect on the BBS in the present study, the SLS was assessed as a second balance assessment and showed statistically significant improvement of 2.4 s post-intervention and at follow-up for the affected leg. However, we have not been able to find any MCID for the SLS. The MDC for the SLS refers to community-dwelling older people and cannot automatically be applied to the affected leg of the stroke population.

One may question whether the current statistically significant improvement of balance after LE-CIMT, together with other studies (6, 19, 20), is a truly clinical meaningful improvement as none of the improvements reach the MCID of BBS detected in the early subacute phase (25). This raised a question as to whether the MCID of BBS detected in the early subacute phase is suitable for comparison with data collected at the chronic stage post-stroke. Subjects in a qualitative study (32), being part of our cohort, reported that they experienced meaningful improvements on balance after participating in LE-CIMT. This may support the argument of meaningful change on balance despite MCID for BBS not being reached. Moreover, the positive effects on balance after LE-CIMT in the present study may play an important role in the improvement of walking ability demonstrated previously in the same cohort (21). Thus, the statistically significant improvements in balance demonstrated in the current study indicate that positive effects after LE-CIMT could be achieved in a real-world setting for stroke survivors even in the chronic stage.

In the present study, we demonstrated significantly increased leg strength with a moderate EF for 1RM (0.54) with significantly increased strength for the affected leg of 10.2 kg. The present result, from a real-world clinical setting, was similar to results from RCTs and Cochrane reviews with various physical training interventions after stroke (17, 32) where the EF was 0.58–0.61 for muscle strength of the paretic leg. Notably, increased leg symmetry was demonstrated by the enhanced ratio between affected/nonaffected limb from 0.61 to 0.72 in the current study. This improvement in leg strength and leg strength symmetry may explain significant improvements in walking capacity and functional mobility our previous study (21). An increase in leg strength for the nonaffected leg was noted even though it was not specifically targeted during the intervention. This may be explained by a generally increased level of activity during and after the therapy. Our results are promising, as community-dwelling people with a chronic stroke have significantly reduced muscle strength in both lower extremities, more so in the affected leg (2, 3). LE-CIMT could therefore be considered a useful intervention to improve muscle strength in both legs and reduce strength asymmetry among stroke survivors in daily clinical practice.

Post-treatment statistically significant improvements on the TUG Manual of 2.7 s despite a small EF (0.14) were demonstrated in our study. This is interesting because, to the best of our knowledge, this is the first time that dual-task ability has been evaluated after CIMT. The improved dual-task ability may have a positive effect on the ability to perform daily activities (4, 15, 33). However, the diff-TUG remained fairly stable, not exceeding 4.5 s, that is, the cut-off value for predicting risk of falls (29). This may suggest that LE-CIMT did not decrease the risk of falling. However, the cut-off value should be used with caution in the present study as it was calculated for community-dwelling older people aged around 80 years. It may be difficult to compare directly with our data from relatively younger persons after stroke. Based on results from other studies (4, 33), our data imply that the improved dual-task ability achieved could contribute to improved functional mobility and decreased risks of fall (5, 34) in our cohort.

The improvements in balance, lower extremity strength, strength symmetry, and dual-task ability were persistent at 3 months post-intervention, which implies residual effects of LE-CIMT. In agreement with our previous study (21), these results underline the value of high-intensity training, such as LE-CIMT, for the maintenance of lower extremity motor function in persons with stroke. The improvements may indicate that the treatment effect of LE-CIMT has transferred to a higher daily activity level, which is a prerequisite for the level of function to remain according to the saying “use-it-or-lose-it” (14, 35). Participants in the study had received routine rehabilitation prior to the 2-week LE-CIMT. Nevertheless, the high-intensity LE-CIMT provided significant additive improvements, with low and moderate EF on balance, leg strength, and dual-task ability. This indicates that LE-CIMT may overcome the saturation or plateau of recovery achieved by routine rehabilitation (36). Our study suggests that high-intensity LE-CIMT may provide additive positive effects on balance, leg strength, and also dual-task ability in the real-word clinical setting.

Compared with the uncertain benefits when using the modified LE-CIMT with relatively low intensity shown in meta-analyses (15, 18), the high-intensity (6 h/day for 2 weeks) LE-CIMT in the current study generated more consistent improvements. Our results support the concept of “more is better” (37, 38). Other key components of the LE-CIMT in this study were mixed training based on shaping strategy, transfer package, and strong encouragement of use of the more affected LE, which has shown significant improvements in LE function (18, 20) in stroke rehabilitation. Meanwhile, many different training methods, such as increased weightbearing, weight shifting, muscle strengthening, physical fitness training (32), and various forms of gait training, were embedded in the LE-CIMT and utilized based on patients’ needs. This may explain the positive effects on balance and leg strength found in the current study as well as the enhanced functional mobility and walking ability previously presented (21).

The present versatile cohort provided the opportunity to investigate which demographic and clinical parameters may affect the impact of LE-CIMT. We found that age was the single factor affecting balance outcomes as assessed by the BBS. In contrast, other parameters, such as sex, type of stroke, affected side, and time span between stroke onset and receiving LE-CIMT, played nonsignificant roles in the impact of LE-CIMT. This indicates that LE-CIMT has good generalizability for stroke survivors with various clinical backgrounds. Caution should be paid to the fact that this cohort was relatively young in comparison with the general stroke population. Participants who received LE-CIMT 1 year or more post-stroke showed similar improvements on assessments compared with those who received the therapy within 6 months post-stroke. Our data suggest that LE-CIMT may result in motor recovery with a considerably broader therapeutic window than the commonly held view of a 6-month therapeutic window for post-stroke recovery (35, 39). Our results indicate that high-intensity LE-CIMT is feasible in a real-world setting.

An obvious strength of the current study is a relatively large sample size with 147 participants, whereas RCT studies often have smaller sample sizes (15). Another strength of the present study is that the outcome measures were assessed before, after, and at the 3-month follow-up. This provided an opportunity to demonstrate the direct effects of the LE-CIMT as well as the durability of improvements. Furthermore, this real-world clinical study demonstrated that high-intensity LE-CIMT was tolerated by many stroke survivors with various characteristics as all participants completed the treatment intervention. Meanwhile, we are aware that this study is a longitudinal cohort study without a control group, which is why the causal relationship between the outcome improvements and LE-CIMT should be discussed with caution. Studies that use robust designs are required to further evaluate the efficacy of LE-CIMT. A potential observer bias is that the same PT conducted the LE-CIMT treatments and assessed the outcomes. However, previous test results were not available at subsequent assessment, which again may decrease the risk of bias. The same PT performing treatment and assessments could, on the other hand, warrant high consistency in this study.

The present study demonstrated that high-intensity LE-CIMT had a positive effect on balance, lower-limb strength, and dual-task ability with at least a 3-month duration of effects among middle-aged participants in subacute and chronic poststroke phases in a real-world outpatient clinical setting.

## References

[1] Langhorne P, Bernhardt J, Kwakkel G. Stroke rehabilitation. Lancet 2011; 377: 1693–1702. 10.1016/S0140-6736(11)60325-5 21571152

[2] Flansbjer UB, Downham D, Lexell J. Knee muscle strength, gait performance, and perceived participation after stroke. Arch Phys Med Rehabil 2006; 87: 974–980. 10.1016/j.apmr.2006.03.008 16813786

[3] Dorsch S, Ada L, Canning CG. Lower limb strength is significantly impaired in all muscle groups in ambulatory people with chronic stroke: a cross-sectional study. Arch Phys Med Rehabil 2016; 97: 522–527. 10.1016/j.apmr.2015.10.106 26615792

[4] Chan PP, Si Tou JI, Tse MM, Ng SS. Reliability and validity of the timed up and go test with a motor task in people with chronic stroke. Arch Phys Med Rehabil 2017; 98: 2213–2220. 10.1016/j.apmr.2017.03.008 28392324

[5] Xu T, Clemson L, O’Loughlin K, Lannin NA, Dean C, Koh G. Risk factors for falls in community stroke survivors: a systematic review and meta-analysis. Arch Phys Med Rehabil 2018; 99: 563–573 e565. 10.1016/j.apmr.2017.06.032 28797618

[6] van Duijnhoven HJ, Heeren A, Peters MA, Veerbeek JM, Kwakkel G, Geurts AC, et al. Effects of exercise therapy on balance capacity in chronic stroke: systematic review and meta-analysis. Stroke 2016; 47: 2603–2610. 10.1161/STROKEAHA.116.013839 27633021

[7] Farrell JW 3rd, Merkas J, Pilutti LA. The effect of exercise training on gait, balance, and physical fitness asymmetries in persons with chronic neurological conditions: a systematic review of randomized controlled trials. Front Physiol 2020; 11: 585765. 10.3389/fphys.2020.585765 33281619 PMC7688661

[8] Taub E, Uswatte G, Mark VW, Morris DM, Barman J, Bowman MH, et al. Method for enhancing real-world use of a more affected arm in chronic stroke: transfer package of constraint-induced movement therapy. Stroke 2013; 44: 1383–1388. 10.1161/STROKEAHA.111.000559 23520237 PMC3703737

[9] Kwakkel G, Veerbeek JM, van Wegen EE, Wolf SL. Constraint-induced movement therapy after stroke. Lancet Neurol 2015; 14: 224–234. 10.1016/S1474-4422(14)70160-7 25772900 PMC4361809

[10] Corbetta D, Sirtori V, Castellini G, Moja L, Gatti R. Constraint-induced movement therapy for upper extremities in people with stroke. Cochrane Database Syst Rev 2015; 2015: CD004433. 10.1002/14651858.CD004433.pub3 26446577 PMC6465192

[11] Dos Anjos S, Morris D, Taub E. Constraint-induced movement therapy for lower extremity function: describing the LE-CIMT protocol. Phys Ther 2020; 100: 698–707. 10.1093/ptj/pzz191 31899495

[12] Hugues A, Di Marco J, Ribault S, Ardaillon H, Janiaud P, Xue Y, et al. Limited evidence of physical therapy on balance after stroke: a systematic review and meta-analysis. PLoS One 2019; 14: e0221700. 10.1371/journal.pone.0221700 31465462 PMC6715189

[13] Socialstyrelsen. Nationella riktlinjer för vård vid stroke 2018. Available from: http://www.socialstyrelsen.se/nationellariktlinjerforstrokesjukvard.

[14] Viana R, Teasell R. Barriers to the implementation of constraint-induced movement therapy into practice. Top Stroke Rehabil 2012; 19: 104–114. 10.1310/tsr1902-104 22436358

[15] Abdullahi A, Truijen S, Umar NA, Useh U, Egwuonwu VA, Van Criekinge T, et al. Effects of lower limb constraint induced movement therapy in people with stroke: a systematic review and meta-analysis. Front Neurol 2021; 12: 638904. 10.3389/fneur.2021.638904 33833730 PMC8021771

[16] Reddy RS, Gular K, Dixit S, Kandakurti PK, Tedla JS, Gautam AP, et al. Impact of constraint-induced movement therapy (CIMT) on functional ambulation in stroke patients: a systematic review and meta-analysis. Int J Environ Res Public Health 2022; 19: 12809. 10.3390/ijerph191912809 36232103 PMC9566465

[17] Veerbeek JM, van Wegen E, van Peppen R, van der Wees PJ, Hendriks E, Rietberg M, et al. What is the evidence for physical therapy poststroke? A systematic review and meta-analysis. PLoS One 2014; 9: e87987. 10.1371/journal.pone.0087987 24505342 PMC3913786

[18] Tedla JS, Gular K, Reddy RS, de Sa Ferreira A, Rodrigues EC, Kakaraparthi VN, et al. Effectiveness of constraint-induced movement therapy (CIMT) on balance and functional mobility in the stroke population: a systematic review and meta-analysis. Healthcare (Basel) 2022; 10: 495. 10.3390/healthcare10030495 35326973 PMC8949312

[19] EMGS ES, Ribeiro TS, da Silva TCC, Costa MFP, Cavalcanti F, Lindquist ARR. Effects of constraint-induced movement therapy for lower limbs on measurements of functional mobility and postural balance in subjects with stroke: a randomized controlled trial. Top Stroke Rehabil 2017; 24: 555–561. 10.1080/10749357.2017.1366011 28859603

[20] Menezes-Oliveira E, da Silva Matuti G, de Oliveira CB, de Freitas SF, Miyuki Kawamura C, Fernandes Lopes JA, et al. Improvement of gait and balance function in chronic post-stroke patients induced by Lower Extremity – Constraint Induced Movement Therapy: a randomized controlled clinical trial. Brain Inj 2024; 38: 559–568. 10.1080/02699052.2024.2328808 38469745

[21] Marklund I, Sefastsson A, Fure B, Klassbo M, Liv P, Stalnacke BM, et al. Lower-extremity constraint-induced movement therapy improved motor function, mobility, and walking after stroke. Eur J Phys Rehabil Med 2023; 59: 136–144. 10.23736/S1973-9087.23.07683-9 36892520 PMC10171361

[22] Dos Anjos SM, Morris DM, Taub E. Constraint-induced movement therapy for improving motor function of the paretic lower extremity after stroke. Am J Phys Med Rehabil 2020; 99: e75–e78. 10.1097/PHM.0000000000001249 31246610

[23] Berg KO, Wood-Dauphinee SL, Williams JI, Maki B. Measuring balance in the elderly: validation of an instrument. Can J Public Health 1992; 83: S7–11. 1468055

[24] Alghadir AH, Al-Eisa ES, Anwer S, Sarkar B. Reliability, validity, and responsiveness of three scales for measuring balance in patients with chronic stroke. BMC Neurol 2018; 18: 141. 10.1186/s12883-018-1146-9 30213258 PMC6136166

[25] Tamura S, Miyata K, Kobayashi S, Takeda R, Iwamoto H. The minimal clinically important difference in Berg Balance Scale scores among patients with early subacute stroke: a multicenter, retrospective, observational study. Top Stroke Rehabil 2022; 29: 423–429. 10.1080/10749357.2021.1943800 34169808

[26] Flansbjer UB, Blom J, Brogardh C. The reproducibility of Berg Balance Scale and the Single-leg Stance in chronic stroke and the relationship between the two tests. PM R 2012; 4: 165–170. 10.1016/j.pmrj.2011.11.004 22306324

[27] Goldberg A, Casby A, Wasielewski M. Minimum detectable change for single-leg-stance-time in older adults. Gait Posture 2011; 33: 737–739. 10.1016/j.gaitpost.2011.02.020 21444208

[28] DeLorme TL. Restoration of muscle power by heavy-resistance exercises. J Bone Joint Surg 1945; 27: 645–667.

[29] Lundin-Olsson L, Nyberg L, Gustafson Y. Attention, frailty, and falls: the effect of a manual task on basic mobility. J Am Geriatr Soc 1998; 46: 758–761. 10.1111/j.1532-5415.1998.tb03813.x 9625194

[30] Peters J, Lauinger A, Mayr M, Ginell K, Abou L. Dual-task assessments for predicting future falls in neurologic conditions: a systematic review. Am J Phys Med Rehabil 2024; 103: 554–560. 10.1097/PHM.0000000000002452 38466165

[31] Sawilowsky SS. New effect size rules of thumb. JMASM (Journal of Modern Applied Statistical Methods) 2009; 8: 26. 10.22237/jmasm/1257035100

[32] Marklund I, Klässbo M, B H. I got knowledge of myself and my prospects for leading an easier life - stroke patients´ experience of training with lower-limb CIMT. Advances in physiotherapy 2010; 12: 134–141. 10.3109/14038190903141048

[33] Silsupadol P, Shumway-Cook A, Lugade V, van Donkelaar P, Chou LS, Mayr U, et al. Effects of single-task versus dual-task training on balance performance in older adults: a double-blind, randomized controlled trial. Arch Phys Med Rehabil 2009; 90: 381–387. 10.1016/j.apmr.2008.09.559 19254600 PMC2768031

[34] Batchelor FA, Hill KD, Mackintosh SF, Said CM, Whitehead CH. Effects of a multifactorial falls prevention program for people with stroke returning home after rehabilitation: a randomized controlled trial. Arch Phys Med Rehabil 2012; 93: 1648–1655. 10.1016/j.apmr.2012.03.031 22503739

[35] Bernhardt J, Hayward KS, Kwakkel G, Ward NS, Wolf SL, Borschmann K, et al. Agreed definitions and a shared vision for new standards in stroke recovery research: the Stroke Recovery and Rehabilitation Roundtable taskforce. Int J Stroke 2017; 12: 444–450. 10.1177/1747493017711816 28697708

[36] Page SJ, Gater DR, Bach YRP. Reconsidering the motor recovery plateau in stroke rehabilitation. Arch Phys Med Rehabil 2004; 85: 1377–1381. 10.1016/j.apmr.2003.12.031 15295770

[37] Lohse KR, Lang CE, Boyd LA. Is more better? Using metadata to explore dose-response relationships in stroke rehabilitation. Stroke 2014; 45: 2053–2058. 10.1161/STROKEAHA.114.004695 24867924 PMC4071164

[38] Clark B, Whitall J, Kwakkel G, Mehrholz J, Ewings S, Burridge J. The effect of time spent in rehabilitation on activity limitation and impairment after stroke. Cochrane Database Syst Rev 2021; 10: CD012612. 10.1002/14651858.CD012612.pub2 34695300 PMC8545241

[39] Murphy TH, Corbett D. Plasticity during stroke recovery: from synapse to behaviour. Nat Rev Neurosci 2009; 10: 861–872. 10.1038/nrn2735 19888284

